# Supplementary material for: Estimation of COVID-19 spread curves integrating global data and borrowing information
Source: PLoS One. 2020 Jul 29;15(7):e0236860. doi: 10.1371/journal.pone.0236860 (PMC7390340; doi:10.1371/journal.pone.0236860)
Supplement: S1 Appendix — (PDF) [file pone.0236860.s001.pdf]

S1 Appendix: Research data for

Estimation of COVID-19 spread curves integrating global data and borrowing information

By SE YOON LEE, BOWEN LEI, and BANI K. MALLICK

Department of Statistics, Texas A&M University, College Station, Texas, 77843, U.S.A.
seyoonlee@stat.tamu.edu bowenlei@stat.tamu.edu bmallick@stat.tamu.edu

S.1 Research data

In this research, we analyzed global COVID-19 data $\{\mathbf{y}_i, \mathbf{x}_i\}_{i=1}^N$, obtained from $N = 40$ countries. (Meanings for the vector notations, $\mathbf{y}_i$ and $\mathbf{x}_i$, will be explained shortly later.) They are listed on Table S.1: each country is contained in the table with form "country name (identifier)", and this identifier also indicates a severity rank, where a lower value indicates a severer status. The order of the ranks thus coincides with the order of the countries named on the y-axis of the Figure 2 in the main manuscript.

We have selected these 40 countries as research targets because they were 40 largest (hence, top 40 severest) in terms of the cumulative numbers for the infected cases in the world on the date May 14th, 2020. In other words, these countries are most severely affected by the COVID-19 in terms of the reported cases on May 14th, 2020.

Table S.1: 40 countries on the research

Country (index i)
US (1), Russia (2), Spain (3), United Kingdom (4), Italy (5),
Brazil (6), France (7), Germany (8), Iran (9), China (10),
India (11), Peru (12), Canada (13), Belgium (14), Saudi Arabia (15),
Netherlands (16), Chile (17), Pakistan (18), Switzerland (19), Portugal (20),
Sweden (21), Qatar (22), Singapore (23), Ireland (24), United Arab Emirates (25),
Poland (26), Japan (27), Israel (28), Romania (29), Austria (30),
Indonesia (31), Philippines (32), South Korea (33), Denmark (34), Egypt (35),
Czechia (36), Norway (37), Australia (38), Malaysia (39), Finland (40)

NOTE: Countries are listed with the form "country name (identifier)". This identifier also represents a severity rank. The rank is measured based on the accumulated number of the confirmed cases on May 14th.

For each country i ($i = 1, \cdots, N$), let y_{it} denotes the number of accumulated confirmed cases for COVID-19 at the t-th time point ($t = 1, \cdots, T$). Here, the time indices $t = 1$ and $t = T$ correspond to the initial and end time points, January 22nd and May 14th, respectively, spanning for $T = 114$ (days). The time series data $\mathbf{y}_i = (y_{i1}, \cdots, y_{it}, \cdots, y_{iT})^\top$ is referred to as an *infection trajectory* for the country i. Infection trajectories for eight countries (US, Russia, UK, Brazil, Germany, China, India, and South Korea) indexed by $i = 1$, 2, 4, 6, 8, 10, 11, and 33, respectively, are displayed in the Figure 1. We collected the data from the Center for Systems Science and Engineering at the Johns Hopkins University.

For each country i, we collected 45 covariates, denoted by $\mathbf{x}_i = (x_{i1}, \cdots, x_{ij}, \cdots, x_{ip})^\top$ ($p = 45$). The 45 predictors can be further grouped by 6 categories: *the 1st category*: general country and population distribution and statistics; *the 2nd category*: general health care resources; *the 3rd category*: tobacco and alcohol use; *the 4th category*: disease and unhealthy prevalence; *the 5th category*: testing and immunization statistics; and *the 6th category*: international health regulations monitoring. The data sources are the World Bank Data (https://data.worldbank.org/-), World Health Organization Data (https://apps.who.int/-), and National Oceanic and Atmospheric Administration (https://www.noaa.gov/-). Detailed explanations for the covariates are described in Section S.2.

S.2 Tables for covariates

Table S.2: Category of covariates.

Category	Covariates (index)
General country and population distribution and statistics	Total_over_65 (1), Female_per (2), Median_age (5), Birth_rate (6), Life_expect_total_60 (14), Dis_to_China (40), Popu_density (44), Tempe_avg (45)
Health care resources	Physician (3), Doc_num_per (12), Hosp_bed (13)
Tobacco and alcohol use	Alcohol_cons_rec (7), Alcohol_cons_unrec (8), Alcohol_consumers_total (9), Heavy_drinking_total (10), Alcohol_death_total (11), Tobacco_smoke (34), Cigarette_smoke (35)
Disease and unhealth prevalence	Underweight_total (4), Blood_glucose (30), Cholesterol (31), Insuf_phy_act (32), Overweight (33), Air_pollution (36), Air_pollution_death (37), Air_pollution_DALYs (38), Tuberculosis_case (39),
Testing and immunization statistics	Dtt_dtp_immun (15), HepB3_immun (16), Hib3_immun (17), MCV1_immun (18), MCV2_immun (19), PCV3_immun (20), Pol3_immun (21), Testing_num (41), Testing_confirm (42), Testing_popu (43)
International Health Regulations monitoring	Zoonotic_Events (22), Food_Safety (23), Laboratory (24), Human_Resources (25), Health_Service_Provision (26), Risk_Communication (27), Points_of_Entry (28), Radiation_Emergencies (29)

NOTE 1: Covariates are listed with the form "predictor name (index)". Predictor names are abbreviated.

NOTE 2: For part of the covariates, the corresponding data available for the public are not for the record of 2020, which may introduce some possible bias in the estimation.

Table S.3: General country and population distribution and statistics.

Covariates (index j)	Explanation
Total_over_65 (1)	Population ages 65 and above (% of total population) in 2018.
Female_per (2)	The percentage of female in the population in 2018.
Median_age (5)	Population median age in 2013.
Birth_rate (6)	Crude birth rate (per 1000 population) in 2013.
Life_expect_total_60 (14)	Life expectancy at age 60 (years) in 2016.
Dis_to_China (40)	Calculated by the R function distm based on the average longitude and latitude.
Popu_density (44)	Population density (people per sq.km of land area) in 2018.
Tempe_avg (45)	The average temperature in February and March in the captain of each country (we choose New York for US and Wuhan for China, due to the severe outbreak in the two cities).

Table S.4: Health care resources.

Covariates (index j)	Explanation
Physician (3)	The number of physicians (per 1000 people) between 2015 and 2018.
Doc_num_per (12)	The number of medical doctors (per 10000 population) in 2016.
Hosp_bed (13)	Average hospital beds (per 10000 population) from 2013 to 2015.

Table S.5: Tobacco and alcohol use.

Covariates (index j)	Explanation
Alcohol_cons_rec (7)	Recorded alcohol consumption per capita (15+) (in litres of pure alcohol), three-year average between 2015 and 2017.
Alcohol_cons_unrec (8)	Unrecorded alcohol consumption per capita (15+) (in litres of pure alcohol) in 2016.
Alcohol_consumers_total (9)	Alcohol consumers past 12 months (those adults who consumed alcohol in the past 12 months) (% of total) in 2016.
Heavy_drinking_total (10)	Age-standardized estimates of the proportion of adults (15+ years) (who have had at least 60 grams or more of pure alcohol on at least one occasion in the past 30 days) in 2016.
Alcohol_death_total (11)	Alcohol-attributable death (% of all-cause deaths in total) in 2016.
Tobacco_smoke (34)	Age-standardized rates of prevalence estimates for daily smoking of any tobacco in adults (15+ years) in 2013.
Cigarette_smoke (35)	Age-standardized rates of prevalence estimates for daily smoking of any cigarette in adults (15+ years) in 2013.

Table S.6: Disease and unhealthy prevalence.

Covariates (index j)	Explanation
Underweight_total (4)	Crude estimate of percent of adults with underweight (BMI $<$ 18.5) in 2016.
Blood_glucose (30)	Age-standardized percent of 18+ population with raised fasting blood glucose ($\geq$7.0 mmol/L or on medication) in 2014.
Cholesterol (31)	Percentage of 25+ population with total cholesterol $\geq$ 240 mg/dl (6.2 mmol/l) in 2008.
Insuf_phy_act (32)	Age-standardized prevalence of insufficient physical activity (% of adults aged 18+) in 2016.
Overweight (33)	Age-standardized prevalence of overweight among adults (BMI $\geq$ 25) (% of adults aged 18+) in 2016.
Air_pollution (36)	Concentrations of fine particulate matter (PM2.5) in 2016.
Air_pollution_death (37)	Age-standardized ambient air pollution attributable death rate (per 100000 population) in 2016.
Air_pollution_DALYs (38)	Age-standardized ambient air pollution attributable Disability-adjusted life year (DALYs) (per 100000 population) in 2016.
Tuberculosis_case (39)	Incidence of tuberculosis (per 100000 population per year) in 2018.

Table S.7: Testing and immunization statistics.

Covariates (index j)	Explanation
Diphtheria tetanus toxoid and pertussis third-dose immunization (15)	Diphtheria tetanus toxoid and pertussis third-dose (DTP3) immunization coverage (% of total 1-year-olds) in 2018.
Hepatitis B third-dose immunization (16)	Hepatitis B third-dose (HepB3) immunization coverage (% of total 1-year-olds) in 2018.
Haemophilus influenzae type B third-dose immunization (17)	Haemophilus influenzae type B third-dose (Hib3) immunization coverage (% of total 1-year-olds) in 2018.
Measles-containing-vaccine first-dose immunization (18)	Measles-containing-vaccine first-dose (MCV1) immunization coverage (% of total 1-year-olds) in 2018.
Measles-containing-vaccine second-dose immunization (19)	Measles-containing-vaccine second-dose (MCV2) immunization coverage (% of total nationally recommended age) in 2018.
Pneumococcal conjugate vaccines third-dose immunization (20)	Pneumococcal conjugate vaccines third-dose (PCV3) immunization coverage (% of total 1-year-olds) in 2018.
Polio third-dose immunization (21)	Polio (Pol3) third-dose immunization coverage (% of total 1-year-olds) in 2018.
Testing_num (41)	The number of COVID-19 testing cases (ourworldindata.org/- collect the data and the data dates are between Febrary and March on several media).
Testing_confirm (42)	The total number of confirmed cases on the same day with testing_num divided by the covariate Testing_num_COVID19 (41).
Testing_popu (43)	The covariate Testing_num_COVID19 (41) divided by covariate Total_popu (2).

Table S.8: International health regulations (IHR) monitoring framework.

Covariates (index j)	Explanation
Zoonotic_Events (22)	Scores that show whether mechanisms for detecting and responding to zoonoses and potential zoonoses are established and functional in 2018.
Food_Safety (23)	Scores that show whether mechanisms are established and functioning for detecting and responding to foodborne disease and food contamination in 2018.
Laboratory (24)	Scores that show the availability of laboratory diagnostic and confirmation services to test for priority health threats in 2018.
Human_Resources (25)	Scores that show the availability of human resources to implement IHR Core Capacity.
Health_Service_Provision (26)	Scores that show an immediate output of the inputs into the health system, such as the health workforce, procurement and supplies, and financing in 2018.
Risk_Communication (27)	Scores that show mechanisms for effective risk communication during a public health emergency are established and functioning in 2018.
Points_of_Entry (28)	Scores that show whether general obligations at point of entry are fulfilled (including for coordination and communication) to prevent the spread of diseases through international traffic in 2018.
Radiation_Emergencies (29)	Scores that show whether mechanisms are established and functioning for detecting and responding to radiological and nuclear emergencies that may constitute a public health event of international concern in 2018.

NOTE 1: The International health regulations, or IHR (2005), represent an agreement between 196 countries including all WHO Member States to work together for global health security. Through IHR, countries have agreed to build their capacities to detect, assess, and report public health events. WHO plays the coordinating role in IHR and, together with its partners, helps countries to build capacities. (https://www.who.int/ihr/about/-)

NOTE 2: IHR monitoring framework was developed, which represents a consensus among technical experts from WHO Member States, technical institutions, partners and WHO. (https://www.who.int/ihr/procedures/-)
