# Supplementary material for: Estimation of COVID-19 spread curves integrating global data and borrowing information
Source: PLoS One. 2020 Jul 29;15(7):e0236860. doi: 10.1371/journal.pone.0236860 (PMC7390340; doi:10.1371/journal.pone.0236860)
Supplement: S2 Appendix — (PDF) [file pone.0236860.s002.pdf]

S2 Appendix: Technical expressions for the three models $\mathcal{M}_1$, $\mathcal{M}_2$, and $\mathcal{M}_3$ for

Estimation of COVID-19 spread curves integrating global data and borrowing information

By SE YOON LEE, BOWEN LEI, and BANI K. MALLICK

Department of Statistics, Texas A&M University, College Station, Texas, 77843, U.S.A.
seyoonlee@stat.tamu.edu bowenlei@stat.tamu.edu bmallick@stat.tamu.edu

S.1 Technical expressions for the three models $\mathcal{M}_1$, $\mathcal{M}_2$, and $\mathcal{M}_3$

Technical expressions for the three models, $\mathcal{M}_1$, $\mathcal{M}_2$, and $\mathcal{M}_3$, compared Subsection **"Benefits from the information borrowing"** in the main manuscript are given as follows:

$\mathcal{M}_1$ is an individual country-based model (non-hierarchical model) that uses infection trajectory for a single country $\mathbf{y} = (y_1, \cdots, y_T)^\top$. The model is given by

$$y_t = f(t; \theta_1, \theta_2, \theta_3, \xi) + \epsilon_t,\ \epsilon_t \sim \mathcal{N}(0, \sigma^2),\ \theta_l \sim \mathcal{N}(\alpha_l, \sigma_l^2),$$
$$\xi \sim \log\ \mathcal{N}(0, 1), (t = 1, \cdots, T,\ l = 1, 2, 3),$$

where $f(t; \theta_1, \theta_2, \theta_3)$ is the Richards growth curve ((1) in the main manuscript), and improper priors are used for error variances and intercept terms as (6).

$\mathcal{M}_2$ is a Bayesian hierarchical model without using covariates, which uses infection trajectories from N countries, $\{\mathbf{y}_i\}_{i=1}^N$. This model is equivalent to BHRM (2) – (6) with removed covariates terms in (3).

$\mathcal{M}_3$ is the BHRM (2) – (6).
