# Supplementary material for: Estimation of COVID-19 spread curves integrating global data and borrowing information
Source: PLoS One. 2020 Jul 29;15(7):e0236860. doi: 10.1371/journal.pone.0236860 (PMC7390340; doi:10.1371/journal.pone.0236860)
Supplement: S3 Appendix — (PDF) [file pone.0236860.s003.pdf]

S3 Appendix: Posterior computation

for

Estimation of COVID-19 spread curves integrating global data and borrowing information

By SE YOON LEE, BOWEN LEI, and BANI K. MALLICK

Department of Statistics, Texas A&M University, College Station, Texas, 77843, U.S.A.
seyoonlee@stat.tamu.edu bowenlei@stat.tamu.edu bmallick@stat.tamu.edu

S.1 Posterior computation

We illustrate a full description of a posterior computation for the BHRM (2) – (6) by using a Markov chain Monte Carlo (MCMC) simulation (Robert and Casella, 2013). To start with, for illustrative purpose, we shall use vectorized notations for the likelihood part (2), regression part (3), and its coefficients part (5):

$$\mathbf{y}_i|\theta_{1i},\theta_{2i},\theta_{3i},\xi_i,\sigma^2 \sim \mathcal{N}_T(\mathbf{f}(\theta_{1i},\theta_{2i},\theta_{3i},\xi_i),\sigma^2\mathbf{I}), \quad (i=1,\cdots,N),$$

$$\boldsymbol{\theta}_l|\alpha_l,\boldsymbol{\beta}_l,\sigma_l^2 \sim \mathcal{N}_N(\mathbf{1}\alpha_l+\mathbf{X}\boldsymbol{\beta}_l,\sigma_l^2\mathbf{I}), \quad (l=1,2,3),$$

$$\boldsymbol{\beta}_l|\tau_l,\boldsymbol{\lambda}_l,\sigma_l^2 \sim \mathcal{N}_p(\mathbf{0},\sigma_l^2\tau_l^2\boldsymbol{\Lambda}_l), \quad (l=1,2,3).$$

The T-dimensional vector $\mathbf{y}_i = (y_{i1},\cdots,y_{it},\cdots,y_{iT})^\top$ $(i=1,\cdots,N)$ is the observed infection trajectory for the country i across the times. The notation $\mathbf{f}(\theta_{1i},\theta_{2i},\theta_{3i},\xi_i)$ $(i=1,\cdots,N)$ is T-dimensional vector that describes the Richard curves across the times:

$$\mathbf{f}(\theta_{1i},\theta_{2i},\theta_{3i},\xi_i) = (f(1;\theta_{1i},\theta_{2i},\theta_{3i},\xi_i),\ldots,f(T;\theta_{1i},\theta_{2i},\theta_{3i},\xi_i))^\top, \quad (i=1,\cdots,N).$$

The vectors $\boldsymbol{\theta}_l = (\theta_{l1},\cdots,\theta_{lN})^\top$ $(l=1,2,3)$ and $\boldsymbol{\xi} = (\xi_1,\cdots,\xi_N)^\top$ are N-dimensional vectors for the four parameters of the Richards curve (1) across the N countries.

The matrix $\mathbf{X}$ is N-by-p design matrix whose i-th row vector is given by the p predictors $\mathbf{x}_i = (x_{i1},\cdots,x_{ip})^\top \in \mathbb{R}^p$, $(i=1,\cdots,N)$. The notation $\mathbf{I}$ stands for an identity matrix. Before implementing, it is recommended that each of column vectors of the design matrix $\mathbf{X}$ is standardized

(Armagan et al., 2013; Tibshirani, 1996): that is, each column vector has been centered, and then columnwisely scaled so that each column vector has mean zero and unit Euclidean norm (l_2-norm).

The p-dimensional vector $\boldsymbol{\beta}_l = (\beta_{l1}, \cdots, \beta_{lp})^\top$ $(l = 1, 2, 3)$ denotes p coefficients from the l-th regression. The vector $\boldsymbol{\lambda}_l = (\lambda_{l1}, \cdots, \lambda_{lp})^\top$ $(l = 1, 2, 3)$ is p-dimensional vector for the local-scale parameters, and the matrix $\boldsymbol{\Lambda}_l$ is p-by-p diagonal matrix $\boldsymbol{\Lambda}_l = \text{diag}(\lambda_{l1}^2, \cdots, \lambda_{lp}^2)$ $(l = 1, 2, 3)$. The τ_l $(l = 1, 2, 3)$ is referred to as the global-scale parameter (Carvalho et al., 2010).

Under the formulation of BHRM (2) – (6), our goal is to sample from the full joint posterior distribution $\pi(\boldsymbol{\theta}_1, \boldsymbol{\theta}_2, \boldsymbol{\theta}_3, \boldsymbol{\xi}, \sigma^2, \Omega_1, \Omega_2, \Omega_3 | \mathbf{y}_{1:N})$ where $\Omega_l = \{\alpha_l, \boldsymbol{\beta}_l, \boldsymbol{\lambda}_l, \tau_l, \sigma_l^2\}$ $(l = 1, 2, 3)$, whose proportional part is given by

$$\left\{ \prod_{i=1}^{N} \mathcal{N}_T(\mathbf{y}_i | \mathbf{f}_i(\theta_{1i}, \theta_{2i}, \theta_{3i}, \xi_i), \sigma^2 \mathbf{I}) \right\} \left\{ \prod_{l=1}^{3} \mathcal{N}_N(\boldsymbol{\theta}_l | \mathbf{1}\alpha_l + \mathbf{X}\boldsymbol{\beta}_l, \sigma_l^2 \mathbf{I}) \mathcal{N}_p(\boldsymbol{\beta}_l | \mathbf{0}, \sigma_l^2 \tau_l^2 \boldsymbol{\Lambda}_l) \pi(\boldsymbol{\lambda}_l) \pi(\tau_l) \pi(\sigma_l^2) \right\}$$
$$\cdot \left\{ \prod_{i=1}^{N} \log\ \mathcal{N}(\xi_i | 0, 1) \right\} \pi(\sigma).$$

To sample from the full joint density, we use a Gibbs sampler (Casella and George, 1992) to exploit conditional independences among the latent variables induced by the hierarchy. The following algorithm describes a straightforward Gibbs sampler

Step 1. Sample $\boldsymbol{\theta}_1$ from its full conditional distribution

$$\pi(\boldsymbol{\theta}_1 | -) \sim \mathcal{N}_N(\boldsymbol{\Sigma}_{\boldsymbol{\theta}_1} \{(1/\sigma^2)\mathbf{r} + (1/\sigma_l^2)(\mathbf{1}\alpha_1 + \mathbf{X}\boldsymbol{\beta}_1)\}, \boldsymbol{\Sigma}_{\boldsymbol{\theta}_1}),$$

where $\boldsymbol{\Sigma}_{\boldsymbol{\theta}_1} = \{(1/\sigma^2)\mathbf{H} + (1/\sigma_l^2)\mathbf{I}\}^{-1} \in \mathbb{R}^{N \times N}$. The matrix $\mathbf{H}$ is N-by-N diagonal matrix $\mathbf{H} = \text{diag}(\|\mathbf{h}(\theta_{21}, \theta_{31}, \xi_1)\|_2^2, \cdots, \|\mathbf{h}(\theta_{2N}, \theta_{3N}, \xi_N)\|_2^2)$, and the vector $\mathbf{r}$ is a N-dimensional vector which is given by $\mathbf{r} = (\mathbf{y}_1^\top \mathbf{h}(\theta_{21}, \theta_{31}, \xi_1), \ldots, \mathbf{y}_N^\top \mathbf{h}(\theta_{2N}, \theta_{3N}, \xi_N))^\top$, where each of the T-dimensional vector $\mathbf{h}(\theta_{2i}, \theta_{3i}, \xi_i)$ $(i = 1, \cdots, N)$ is obtained by

$$\mathbf{h}(\theta_{2i}, \theta_{3i}, \xi_i) = (h(1; \theta_{2i}, \theta_{3i}, \xi_i), \ldots, h(T; \theta_{2i}, \theta_{3i}, \xi_i))^\top,$$

where $h(t; \theta_2, \theta_3, \xi) = [1 + \xi \cdot \exp\{-\theta_2 \cdot (t - \theta_3)\}]^{-1/\xi}$.

Step 2. Sample θ_{2i} and θ_{3i}, $i = 1, \cdots, N$, independently from their full conditional distributions. Proportional parts of the distributions are given by

$$\pi(\theta_{2i} | -) \propto \exp\left(-\frac{1}{2\sigma^2} \|\mathbf{y}_i - \mathbf{f}(\theta_{1i}, \theta_{2i}, \theta_{3i}, \xi_i)\|_2^2 - \frac{1}{2\sigma_2^2}(\theta_{2i} - \alpha_2 - \mathbf{x}_i^\top \boldsymbol{\beta}_2)^2 \right),$$
$$\pi(\theta_{3i} | -) \propto \exp\left(-\frac{1}{2\sigma^2} \|\mathbf{y}_i - \mathbf{f}(\theta_{1i}, \theta_{2i}, \theta_{3i}, \xi_i)\|_2^2 - \frac{1}{2\sigma_3^2}(\theta_{3i} - \alpha_3 - \mathbf{x}_i^\top \boldsymbol{\beta}_3)^2 \right).$$

Here, $\|\cdot\|_2$ indicates the l_2-norm. Note that the two conditional densities are not known in

closed forms because two parameters, θ_{2i} and θ_{3i}, participate to the function $\mathbf{f}(\theta_{1i}, \theta_{2i}, \theta_{3i}, \xi_i)$ in a nonlinear way. We use the Metropolis algorithm (Andrieu et al., 2003) with Gaussian proposal densities within this Gibbs sampler algorithm.

Step 3. Sample ξ_i, $i = 1, \cdots, N$, independently from its full conditional distribution. Proportional parts of the distributions are given by

$$\pi(\xi_i|-) \propto \exp\left(- \frac{1}{2\sigma^2} \|\mathbf{y}_i - \mathbf{f}(\theta_{1i}, \theta_{2i}, \theta_{3i}, \xi_i)\|_2^2 \right) \cdot \log\ \mathcal{N}(\xi_i|0, 1). \tag{S.1}$$

Note that the density (S.1) is not expressed in a closed form distribution. Because the shape parameter ξ_i is supported on $(0, \infty)$ and participates in the Richards curve (1) as an exponent, sampling from the density needs a delicate care, where by we employed the elliptical slice sampler (Murray et al., 2010).

Step 4. Sample σ^2 from its full conditional distribution

$$\pi(\sigma^2|-) \sim \mathcal{IG}\left(\frac{NT}{2}, \frac{1}{2} \sum_{i=1}^{N} \|\mathbf{y}_i - \mathbf{f}(\theta_{1i}, \theta_{2i}, \theta_{3i}, \xi_i)\|_2^2 \right).$$

Step 5. Sample α_l, $l = 1, 2, 3$, independently from their full conditional distributions

$$\pi(\alpha_l|-) \sim \mathcal{N}_1(\mathbf{1}^\top(\boldsymbol{\theta}_l - \mathbf{X}\boldsymbol{\beta}_l)/N, \sigma_l^2/N).$$

Step 6. Sample $\boldsymbol{\beta}_l$, $l = 1, 2, 3$, independently from conditionally independent posteriors

$$\pi(\boldsymbol{\beta}_l|-) \sim \mathcal{N}_p(\boldsymbol{\Sigma}_{\boldsymbol{\beta}_l} \mathbf{X}^\top (\boldsymbol{\theta}_l - \mathbf{1}\alpha_l), \sigma_l^2 \boldsymbol{\Sigma}_{\boldsymbol{\beta}_l}),$$

where $\boldsymbol{\Sigma}_{\boldsymbol{\beta}_l} = [\mathbf{X}^\top\mathbf{X} + \Lambda_{*l}^{-1}]^{-1} \in \mathbb{R}^{p\times p}$, $\boldsymbol{\Lambda}_l = \mathrm{diag}(\lambda_{l1}^2, \cdots, \lambda_{lp}^2) \in \mathbb{R}^{p\times p}$, and $\boldsymbol{\Lambda}_{*l} = \tau_l^2 \boldsymbol{\Lambda}_l$.

Step 7. Sample λ_{lj}, $l = 1, 2, 3$, $j = 1, \cdots, p$, independently from conditionally independent posteriors

$$\pi(\lambda_{lj}|-) \sim \mathcal{N}(\beta_{lj}|0, \sigma_l^2 \tau_l^2 \lambda_{lj}^2) \cdot \{1/(1 + \lambda_{lj}^2)\}.$$

Note that the densities $\pi(\lambda_{lj}|-)$ $(l = 1, 2, 3,\ j = 1, \cdots, p)$ are not expressed in closed forms: we use the slice sampler (Neal, 2003).

Step 8. Sample τ_l, $l = 1, 2, 3$, independently from conditionally independent posteriors

$$\pi(\tau_l|-) \sim \mathcal{N}_p(\boldsymbol{\beta}_l|\mathbf{0}, \sigma_l^2 \tau_l^2 \boldsymbol{\Lambda}_l) \cdot \{1/(1 + \tau_l^2)\}.$$

Note that the densities $\pi(\tau_l|-)$ $(l = 1, 2, 3)$ are not expressed in closed forms: we use the slice sampler (Neal, 2003).

Step 9. Sample σ_l^2, $l = 1, 2, 3$, independently from their full conditionally distributions

$$\pi(\sigma_l^2|-) \sim \mathcal{IG}\left(\frac{N+p}{2}, \frac{\|\boldsymbol{\theta}_l - \mathbf{1}\alpha_l - \mathbf{X}\boldsymbol{\beta}_l\|_2^2 + \boldsymbol{\beta}_l^\top \boldsymbol{\Lambda}_{*l}^{-1}\boldsymbol{\beta}_l}{2}\right).$$

Elliptical slice sampler for Step 3

To start with we shall use the variable change $(\eta = \log \xi)$ to the right hand side of (S.1):

$$\pi(\eta_i|-) \propto \mathcal{L}(\eta_i) \cdot \mathcal{N}(\eta_i|0, 1), \quad (i = 1, \cdots, N), \tag{S.2}$$

such that $\mathcal{L}(\eta_i) = \exp\{-\|\mathbf{y}_i - \mathbf{f}(\theta_{1i}, \theta_{2i}, \theta_{3i}, e^{\eta_i})\|_2^2/(2\sigma^2)\}$ corresponds to a likelihood part.

Now, we use the elliptical slice sampler (ESS) (Murray et al., 2010; Nishihara et al., 2014) to sample from $\eta_i^{(s+1)} \sim \pi(\eta_i|-)$ (S.2) $(i = 1, \cdots, N)$ that exploits the Gaussian prior measure. Conceptually, ESS and the Metropolis-Hastings (MH) algorithm (Chib and Greenberg, 1995) are similar: both methods are comprised of two steps: *proposal step* and *criterion step*. A difference between the two algorithms arises in the *criterion step*. If the new candidate does not pass the criterion, then MH takes the current state as the next state: whereas, ESS re-proposes a new candidate until rejection does not take place, rendering the algorithm rejection-free. Further information for ESS is referred to the original paper (Murray et al., 2010). After ESS has been employed, the realized η_i needs to be transformed back through $\xi = e^{\eta}$. Algorithm 1 illustrates the full description of algorithms: to avoid notation clutter, the index i is omitted.

Algorithm 1: Elliptical slice sampler to sample from $\pi(\xi|-)$

Circumstance : At the ***Step 3*** of the s-th iteration of the Gibbs sampler.
Input : Current state $\xi^{(s)}$.
Output : A new state $\xi^{(s+1)}$.

1. Variable change ($\eta = \log \xi$): $\eta^{(s)} = \log\ \xi^{(s)}$.
2. Implement elliptical slice sampler;
   a. Choose an ellipse : $\nu \sim \mathcal{N}_1(0, 1)$.
   b. Define a criterion function:
   $$\alpha(\eta, \eta^{(s)}) = \min\{\mathcal{L}(\eta)/\mathcal{L}(\eta^{(s)}), 1\} : \mathbb{R} \to [0, 1],$$
   where $\mathcal{L}(\eta) = \exp\{-\|\mathbf{y} - \mathbf{f}(\theta_1, \theta_2, \theta_3, e^\eta)\|_2^2/(2\sigma^2)\}$.
   c. Choose a threshold and fix: $u \sim \mathcal{U}[0, 1]$.
   d. Draw an initial proposal η^*:
   $$\phi \sim \mathcal{U}(-\pi, \pi]$$
   $$\eta^* = \eta^{(s)} \cos\ \phi + \nu \sin\ \phi$$
   e. **if** ($u < \alpha(\eta^*, \eta^{(s)})$) { $\eta^{(s+1)} = \eta^*$ } **else** {
      Define a bracket : $(\phi_{\min}, \phi_{\max}] = (-\pi, \pi]$.
      while ($u \geq \alpha(\eta^*, \eta^{(s)})$) {
         Shrink the bracket and try a new point :
         if ($\phi > 0$) $\phi_{\max} = \phi$ **else** $\phi_{\min} = \phi$
            $\phi \sim \mathcal{U}(\phi_{\min}, \phi_{\max}]$
            $\eta^* = \eta^{(s)} \cos\ \phi + \nu \sin\ \phi$
         }
      $\eta^{(s+1)} = \eta^*$
      }

4. Variable change ($\xi = e^\eta$): $\xi^{(s+1)} = \exp\ \eta^{(s+1)}$.

References

Andrieu, C., N. De Freitas, A. Doucet, and M. I. Jordan (2003). An introduction to mcmc for machine learning. *Machine learning 50*(1-2), 5–43.

Armagan, A., D. B. Dunson, and J. Lee (2013). Generalized double pareto shrinkage. *Statistica Sinica 23*(1), 119.

Carvalho, C. M., N. G. Polson, and J. G. Scott (2010). The horseshoe estimator for sparse signals. *Biometrika 97*(2), 465–480.

Casella, G. and E. I. George (1992). Explaining the gibbs sampler. *The American Statistician 46*(3), 167–174.

Chib, S. and E. Greenberg (1995). Understanding the metropolis-hastings algorithm. *The american statistician 49*(4), 327–335.

Murray, I., R. Prescott Adams, and D. J. MacKay (2010). Elliptical slice sampling.

Neal, R. M. (2003). Slice sampling. *Annals of statistics*, 705–741.

Nishihara, R., I. Murray, and R. P. Adams (2014). Parallel mcmc with generalized elliptical slice sampling. *The Journal of Machine Learning Research 15*(1), 2087–2112.

Robert, C. and G. Casella (2013). *Monte Carlo statistical methods.* Springer Science & Business Media.

Tibshirani, R. (1996). Regression shrinkage and selection via the lasso. *Journal of the Royal Statistical Society: Series B (Methodological) 58*(1), 267–288.
