# Supplementary material for: Estimation of COVID-19 spread curves integrating global data and borrowing information
Source: PLoS One. 2020 Jul 29;15(7):e0236860. doi: 10.1371/journal.pone.0236860 (PMC7390340; doi:10.1371/journal.pone.0236860)
Supplement: S4 Appendix — (PDF) [file pone.0236860.s004.pdf]

S4 Appendix for

Estimation of COVID-19 spread curves integrating global data and borrowing information

By SE YOON LEE, BOWEN LEI, and BANI K. MALLICK

Department of Statistics, Texas A&M University, College Station, Texas, 77843, U.S.A.
seyoonlee@stat.tamu.edu bowenlei@stat.tamu.edu bmallick@stat.tamu.edu

S.1 Infection trajectories for the top 20 countries

The section includes extrapolated infection trajectories for the top 20 countries that are most severely affected by the COVID-19.

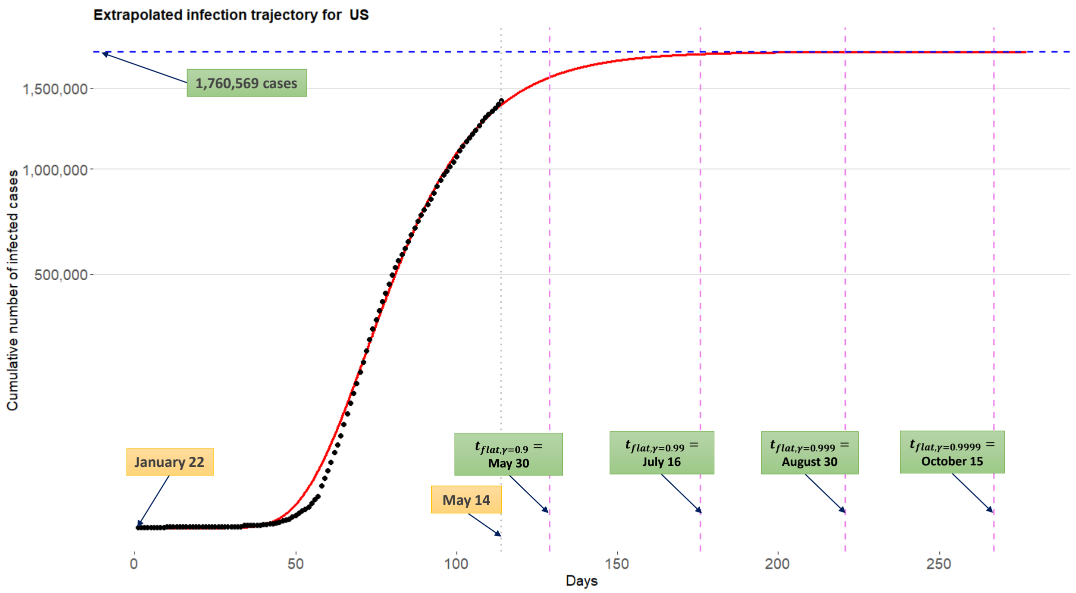

Figure S.1: Extrapolated infection trajectory for the US based on the model $\mathcal{M}_3$.

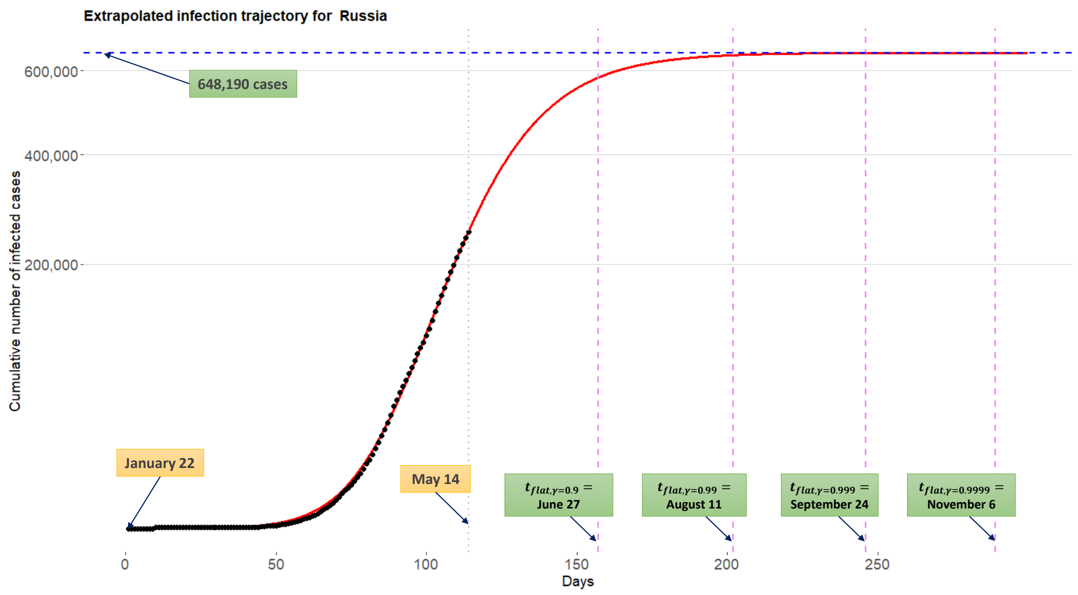

Figure S.2: Extrapolated infection trajectory for the Russia based on the model $\mathcal{M}_3$.

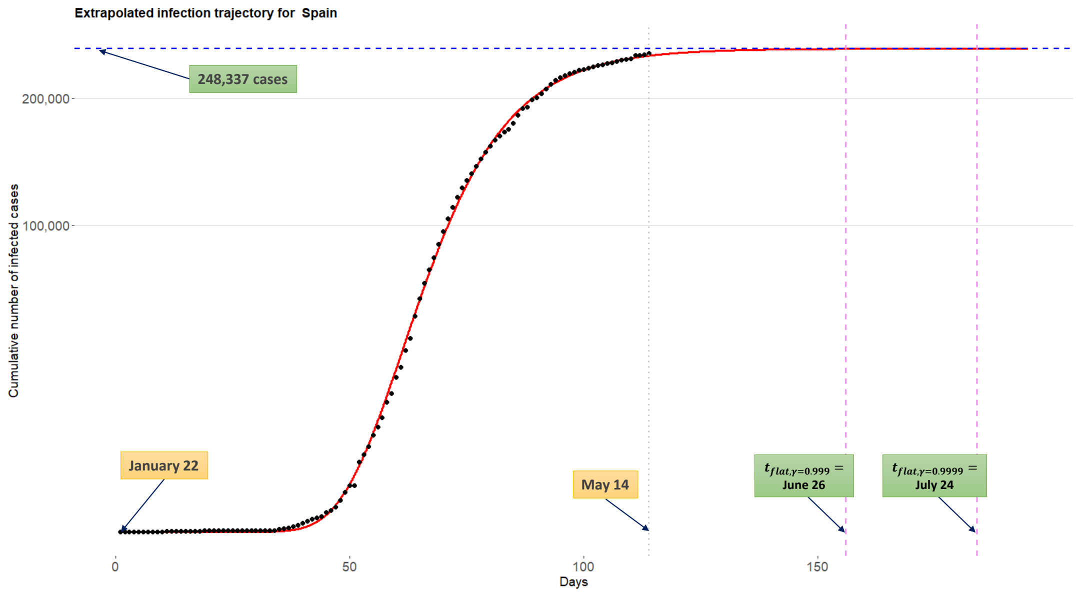

Figure S.3: Extrapolated infection trajectory for the Spain based on the model $\mathcal{M}_3$.

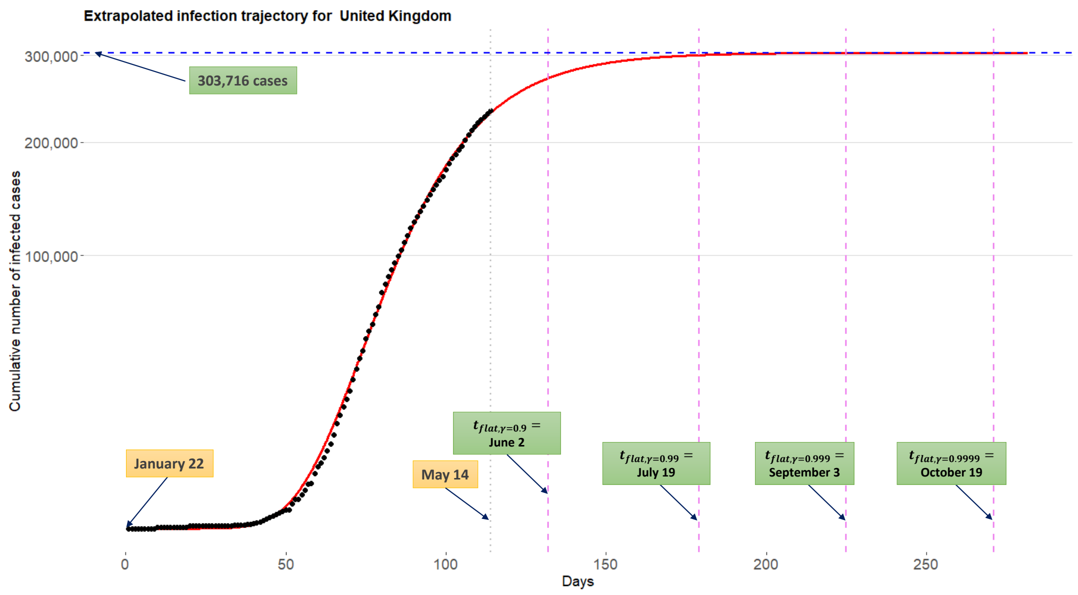

Figure S.4: Extrapolated infection trajectory for the United Kingdom based on the model $\mathcal{M}_3$.

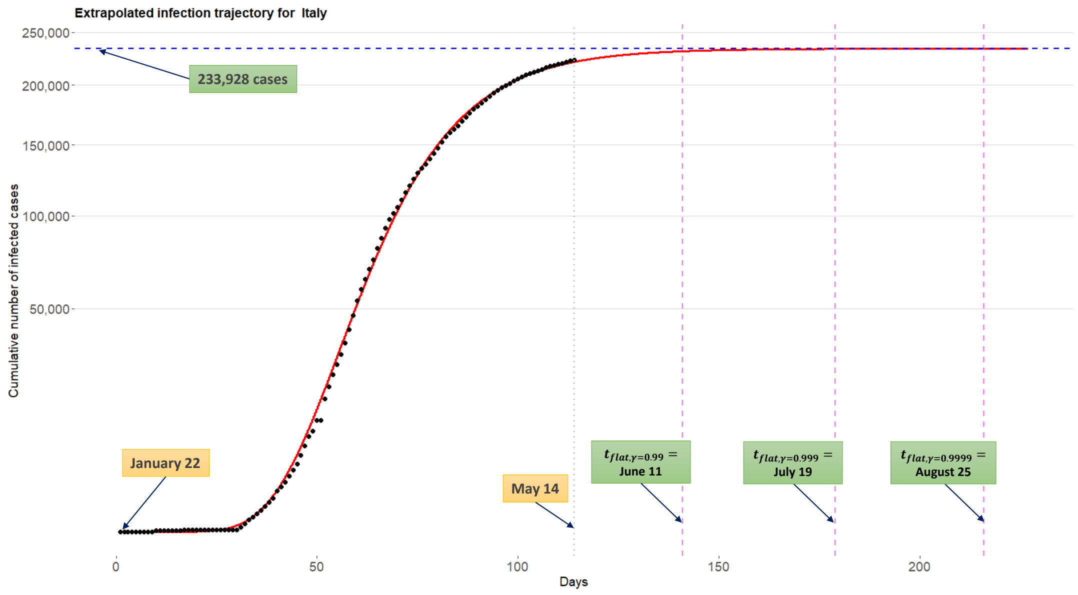

Figure S.5: Extrapolated infection trajectory for the Italy based on the model $\mathcal{M}_3$.

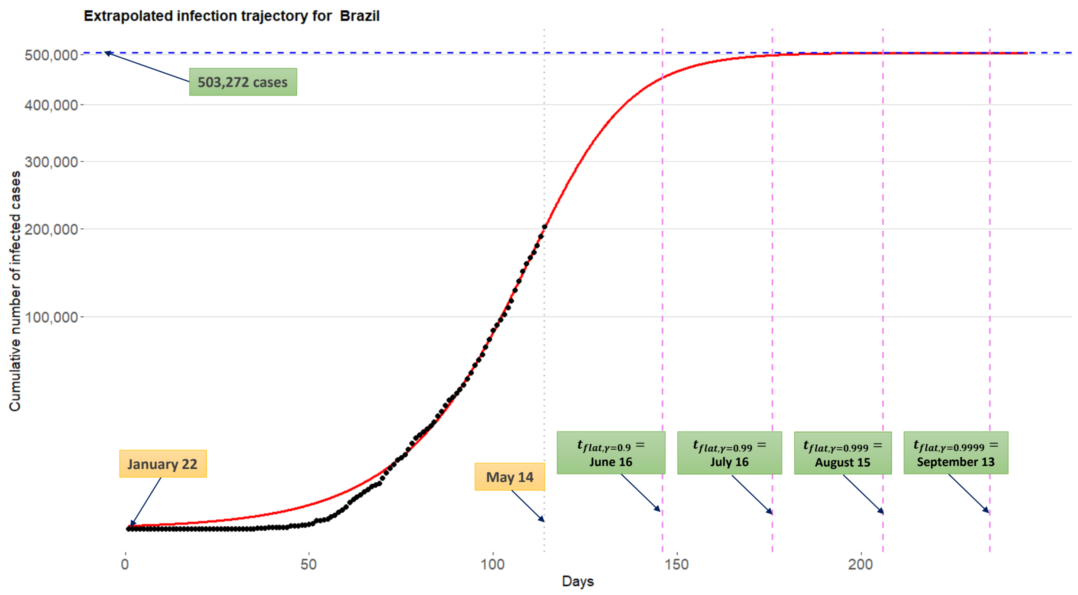

Figure S.6: Extrapolated infection trajectory for the Brazil based on the model $\mathcal{M}_3$.

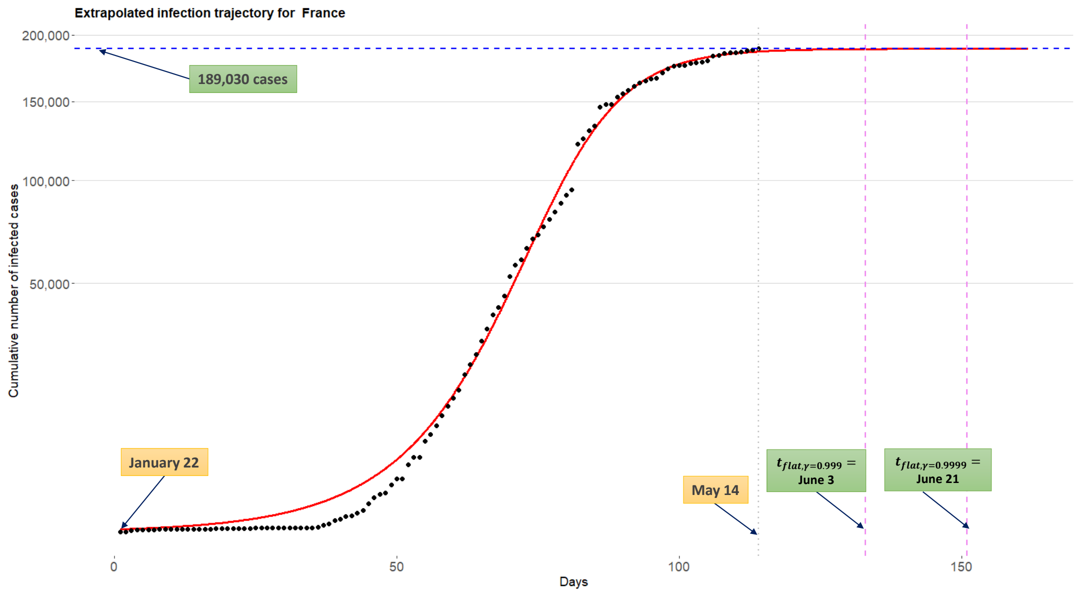

Figure S.7: Extrapolated infection trajectory for the France based on the model $\mathcal{M}_3$.

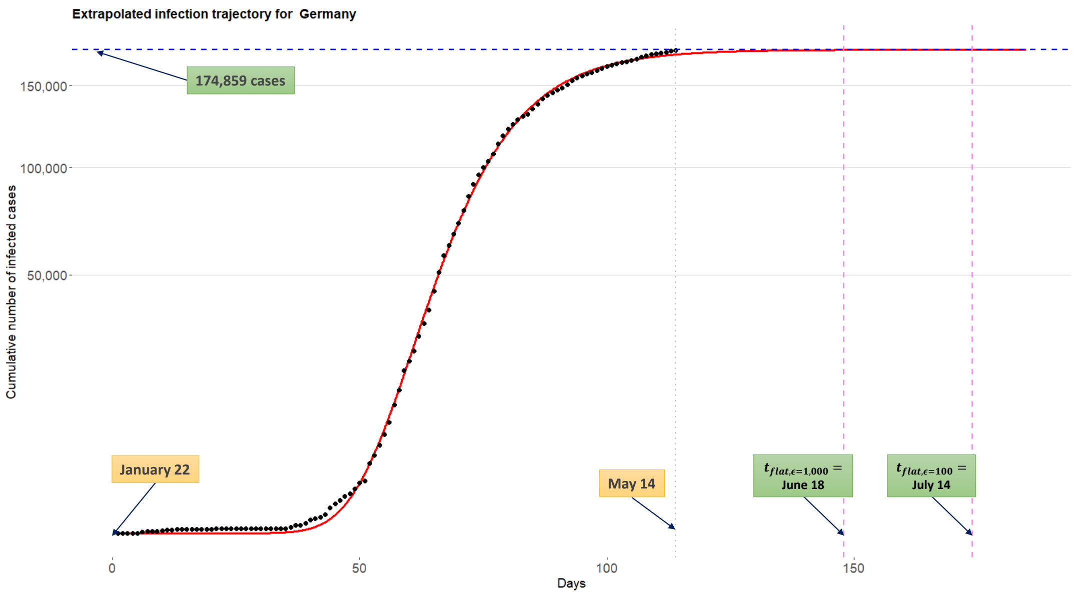

Figure S.8: Extrapolated infection trajectory for the Germany based on the model $\mathcal{M}_3$.

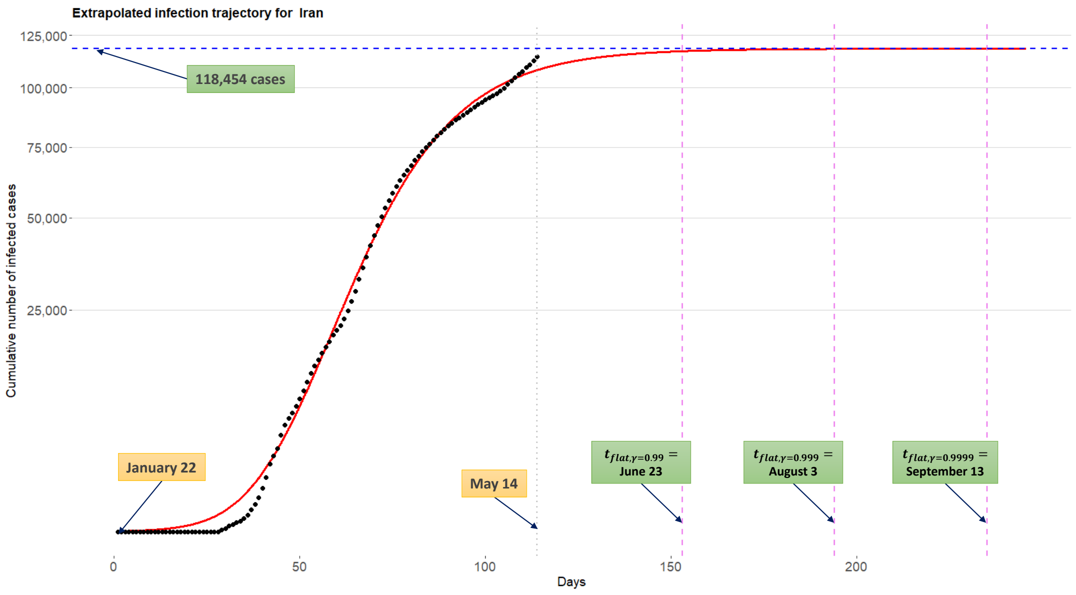

Figure S.9: Extrapolated infection trajectory for the Iran based on the model $\mathcal{M}_3$.

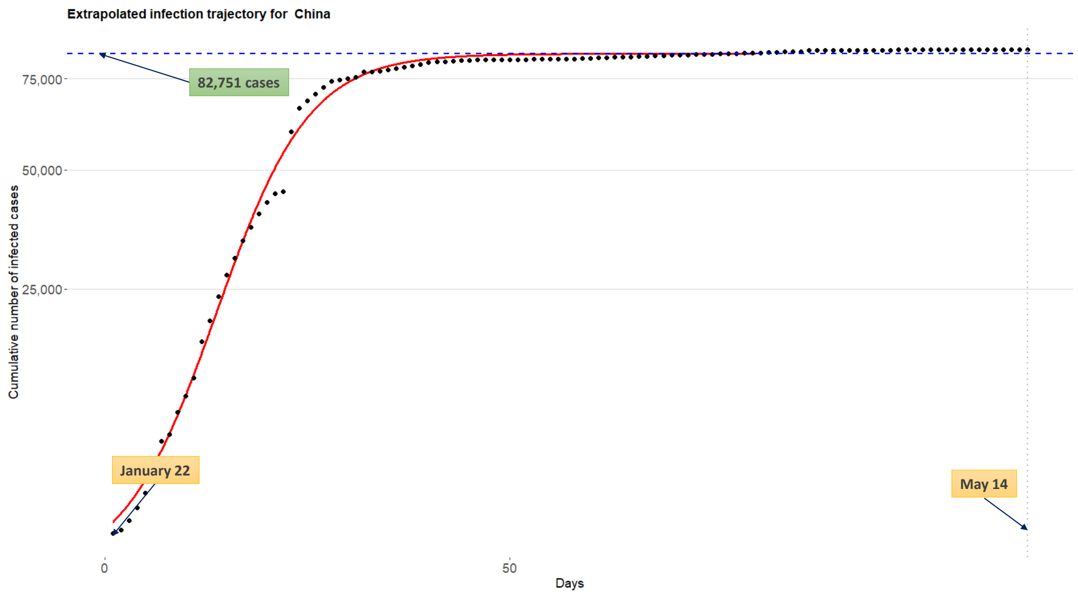

Figure S.10: Extrapolated infection trajectory for the China based on the model $\mathcal{M}_3$.

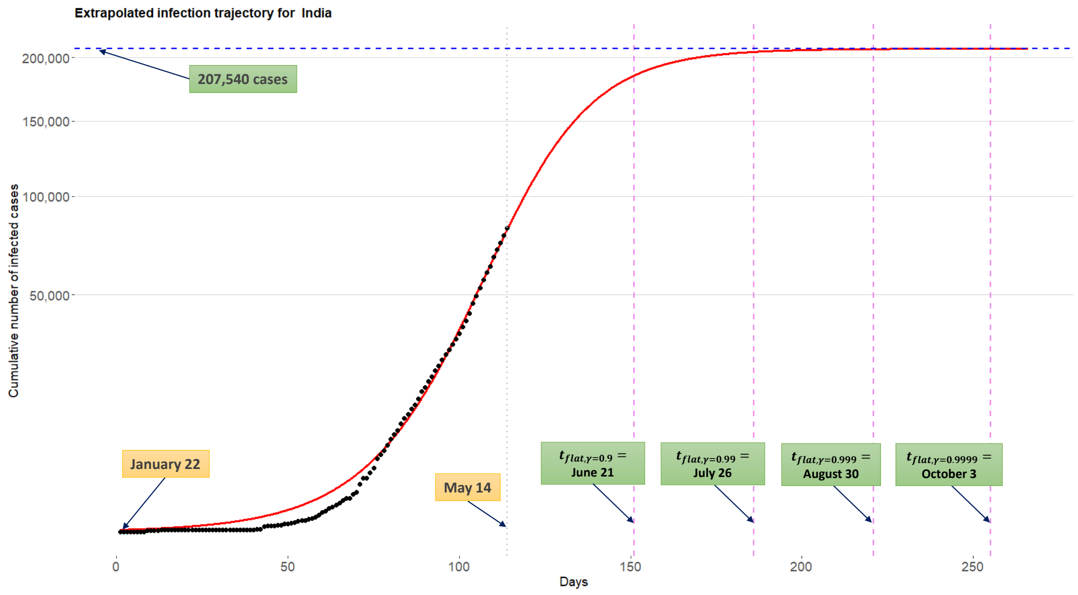

Figure S.11: Extrapolated infection trajectory for the India based on the model $\mathcal{M}_3$.

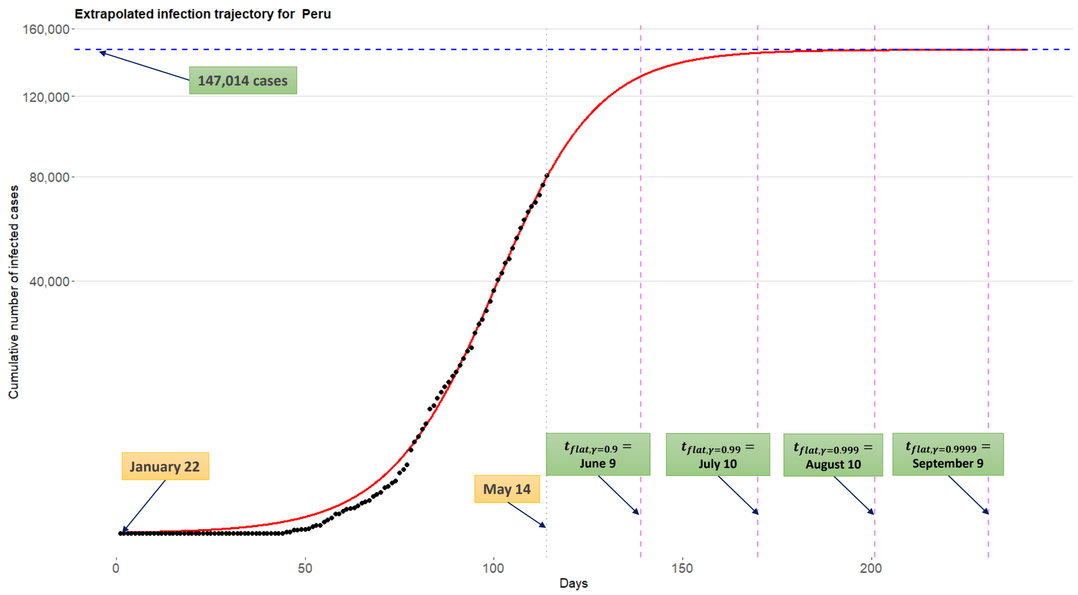

Figure S.12: Extrapolated infection trajectory for the Peru based on the model $\mathcal{M}_3$.

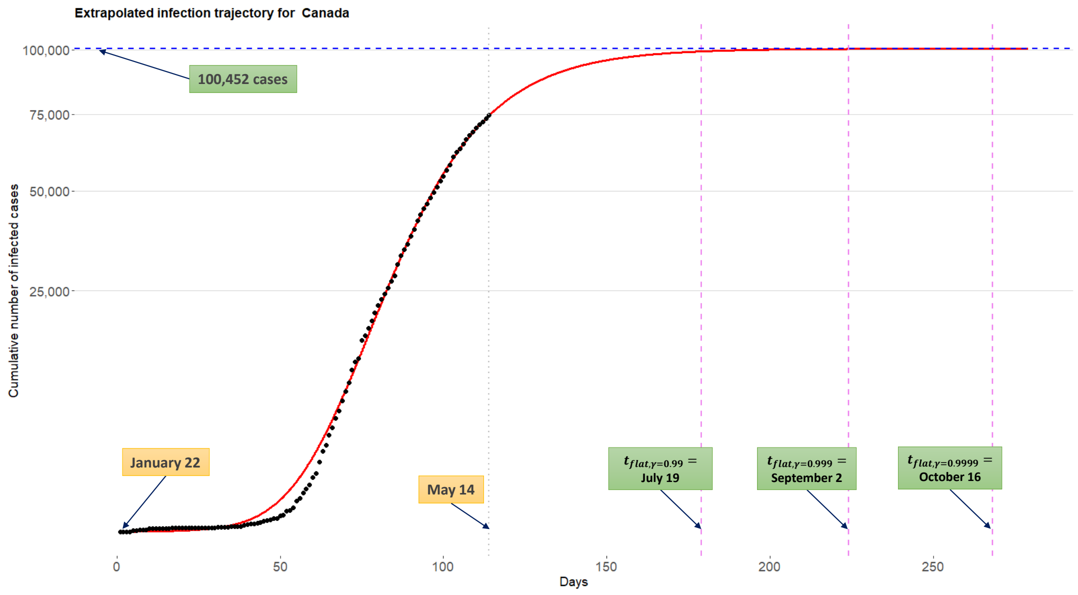

Figure S.13: Extrapolated infection trajectory for the Canada based on the model $\mathcal{M}_3$.

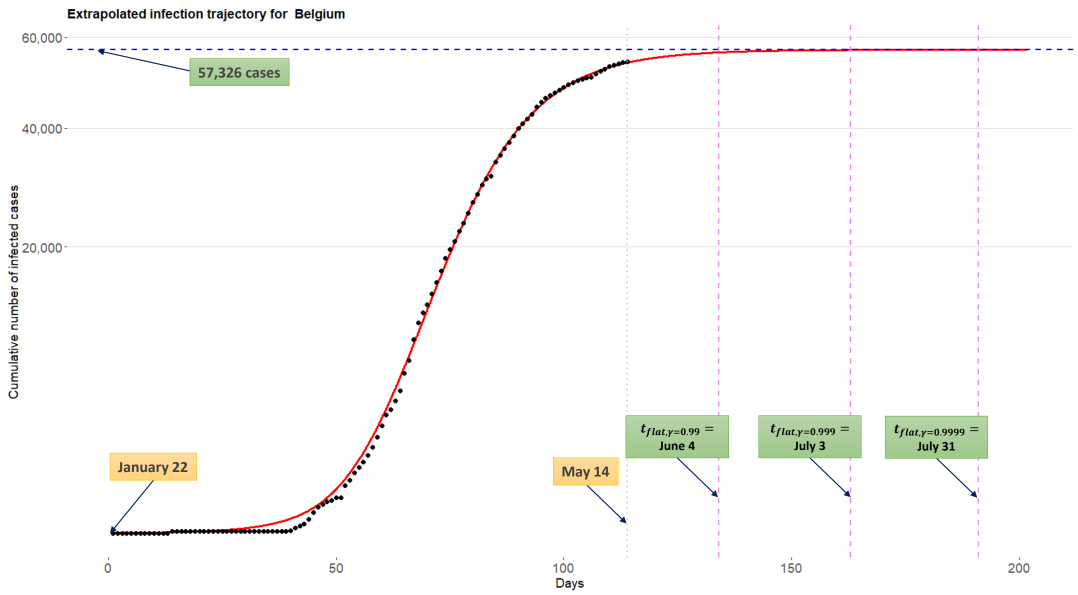

Figure S.14: Extrapolated infection trajectory for the Belgium based on the model $\mathcal{M}_3$.

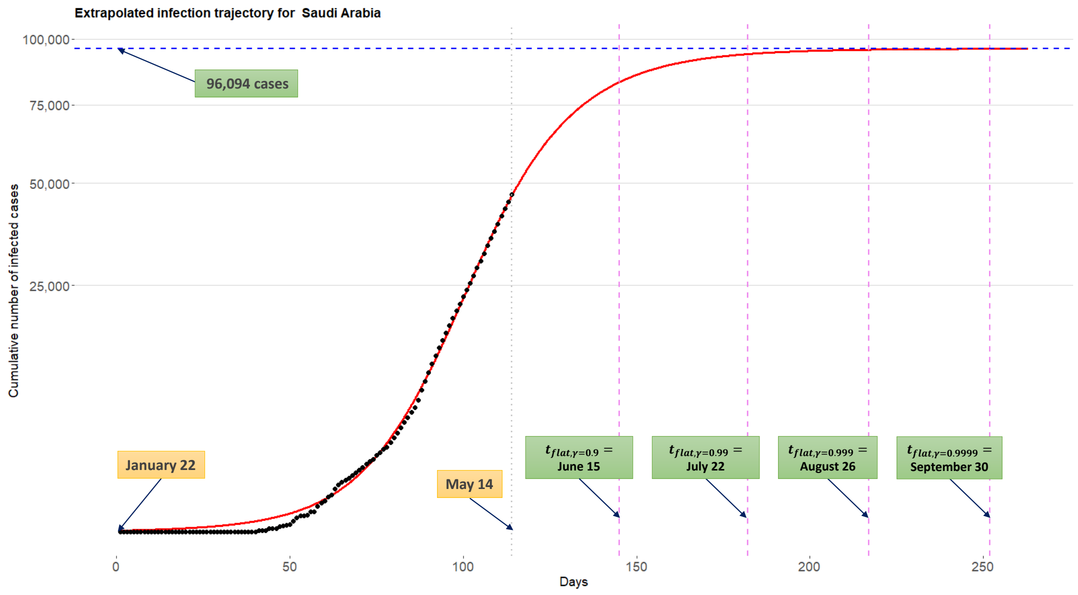

Figure S.15: Extrapolated infection trajectory for the Saudi Arabia based on the model $\mathcal{M}_3$.

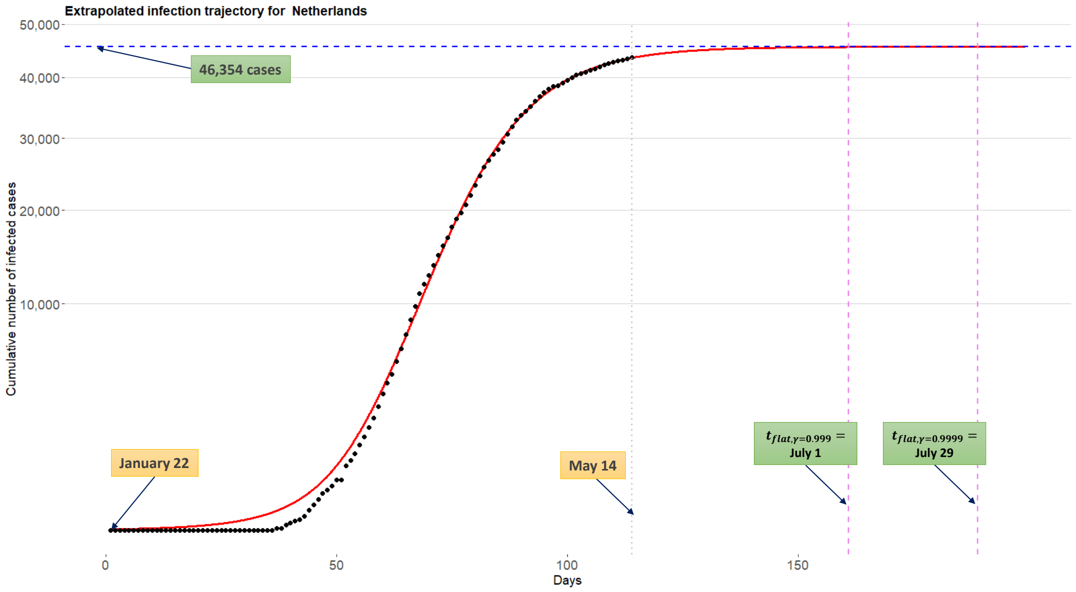

Figure S.16: Extrapolated infection trajectory for the Netherlands based on the model $\mathcal{M}_3$.

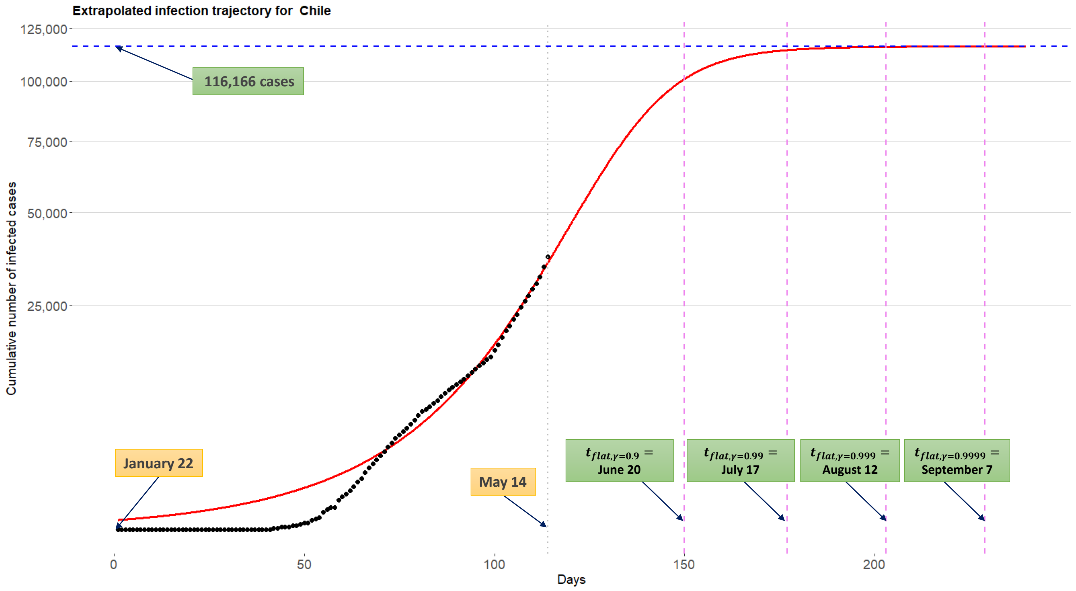

Figure S.17: Extrapolated infection trajectory for the Chile based on the model $\mathcal{M}_3$.

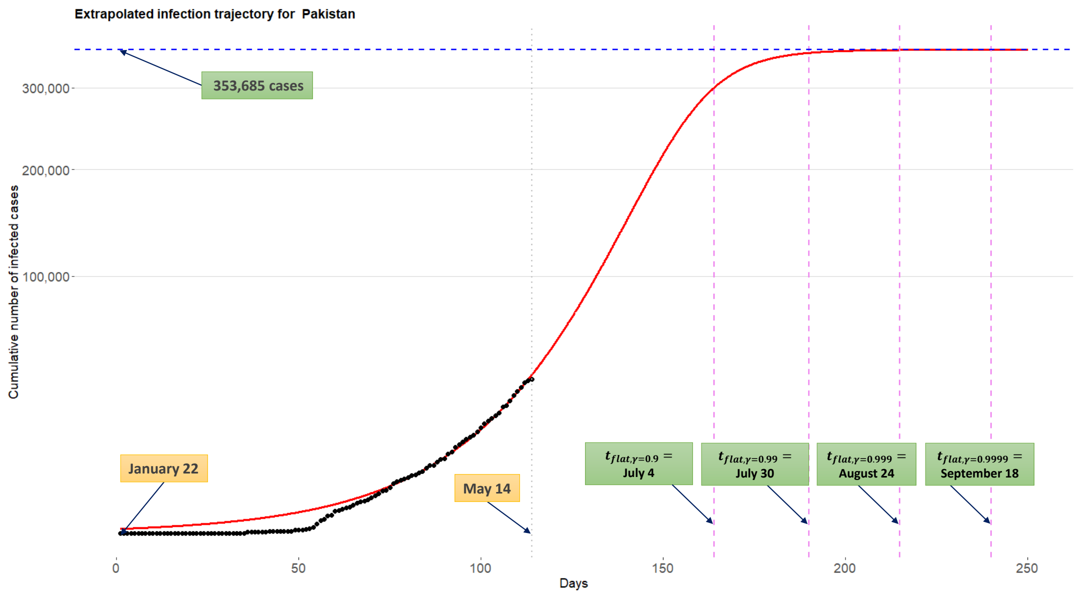

Figure S.18: Extrapolated infection trajectory for the Pakistan based on the model $\mathcal{M}_3$.

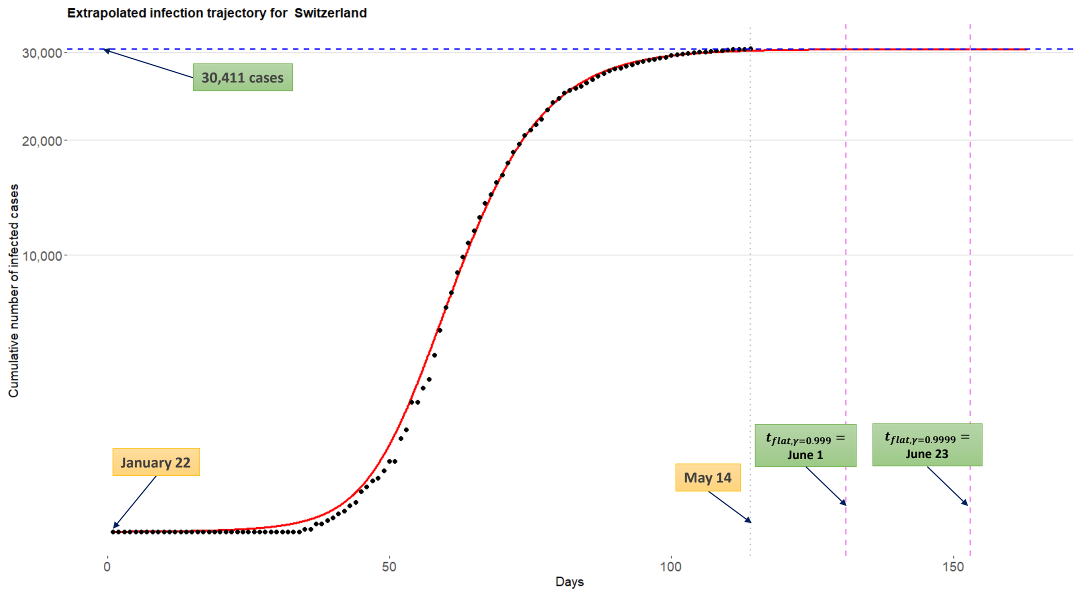

Figure S.19: Extrapolated infection trajectory for the Switzerland based on the model $\mathcal{M}_3$.

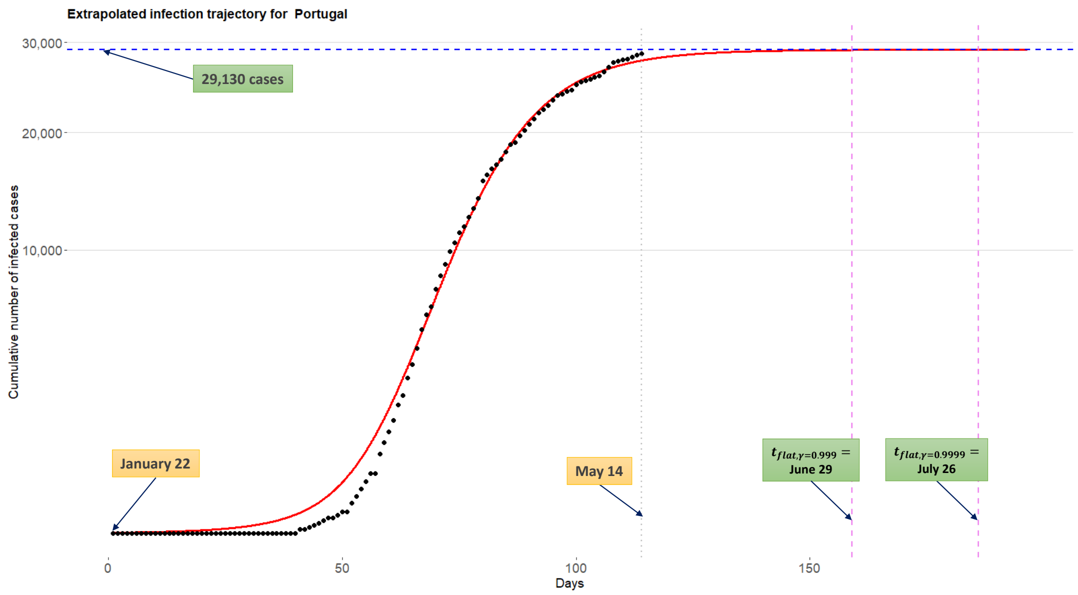

Figure S.20: Extrapolated infection trajectory for the Portugal based on the model $\mathcal{M}_3$.
