# Supplementary material for: Estimation of COVID-19 spread curves integrating global data and borrowing information
Source: PLoS One. 2020 Jul 29;15(7):e0236860. doi: 10.1371/journal.pone.0236860 (PMC7390340; doi:10.1371/journal.pone.0236860)
Supplement: S1 File — (ZIP) [file pone.0236860.s005.zip › S1_File/Estimation_of_COVID-19_spread_curves.html]

Estimation of COVID-19 spread curves


# Estimation of COVID-19 spread curves

***NOTE:*** Objective of this code is to estimate the COVID-19 spread curves based on Bayesian hierarchical Richard model (BHRM), which can integrate global data and borrow information between countries.

Load the time series data and covariates.

```
path <- getwd()
setwd(paste0(path,"/dataset"))
time_series_data <- read.csv("time_series_data.csv")
Y = as.matrix(time_series_data[,-c(1,2)])
design_matrix <- read.csv("design_matrix.csv")
X = as.matrix(design_matrix[,-c(1,2)])
```

Source a function for the BHRM with covariate and run the model.

```
setwd(paste0(path,"/functions"))
source(file = "BHRM_cov.R")
library("compiler")
BHRM_cov= cmpfun(BHRM_cov)

# Execution
seed.no = 1 ; burn = 10000 ; nmc = 5000 ; thin = 10
varrho = 0
pro.var.theta.2 = 0.0002 ; pro.var.theta.3 = 0.05
mu = 0 ; rho.sq = 1
{
  res_cov = BHRM_cov(Y = Y, X = X, seed.no = seed.no, burn = burn, nmc = nmc, thin = thin, 
                 varrho = varrho, pro.var.theta.2 = pro.var.theta.2, pro.var.theta.3 = pro.var.theta.3, mu = mu, rho.sq = rho.sq)  
}
```

Draw the extrapolated infection trajectories and flat time points

```
setwd(paste0(path,"/functions"))
source("visualizations_individual.R")
source("flat_time_point.R")
```

## US

Draw the figure for US:

```
output <- visualizations_individual(1, res_cov, time_series_data)
output$figure
```

The epidemic size for the US:

```
output$epidemic_size
```

```
## [1] 1761992
```

The flat time points for the US (\(\epsilon=0.9,0.99,0.999,0.9999\)):

```
output$flat_time
```

```
## [1] "2020-05-30" "2020-07-16" "2020-08-30" "2020-10-15"
```

## Russia

Draw the figure for Russia:

```
output <- visualizations_individual(2, res_cov, time_series_data)
output$figure
```

The epidemic size for the Russia:

```
output$epidemic_size
```

```
## [1] 591661
```

The flat time points for the Russia (\(\epsilon=0.9,0.99,0.999,0.9999\)):

```
output$flat_time
```

```
## [1] "2020-06-22" "2020-08-02" "2020-09-12" "2020-10-22"
```

## United Kingdom

Draw the figure for United Kingdom:

```
output <- visualizations_individual(4, res_cov, time_series_data)
output$figure
```

The epidemic size for the United Kingdom:

```
output$epidemic_size
```

```
## [1] 299259
```

The flat time points for the United Kingdom (\(\epsilon=0.9,0.99,0.999,0.9999\)):

```
output$flat_time
```

```
## [1] "2020-06-01" "2020-07-16" "2020-08-30" "2020-10-14"
```

## Brazil

Draw the figure for United Brazil:

```
output <- visualizations_individual(6, res_cov, time_series_data)
output$figure
```

The epidemic size for the Brazil:

```
output$epidemic_size
```

```
## [1] 497315
```

The flat time points for the Brazil (\(\epsilon=0.9,0.99,0.999,0.9999\)):

```
output$flat_time
```

```
## [1] "2020-06-15" "2020-07-14" "2020-08-11" "2020-09-08"
```
