# Supplementary figures and images for: Estimation of COVID-19 spread curves integrating global data and borrowing information
Source: PLoS One. 2020 Jul 29;15(7):e0236860. doi: 10.1371/journal.pone.0236860 (PMC7390340; doi:10.1371/journal.pone.0236860)

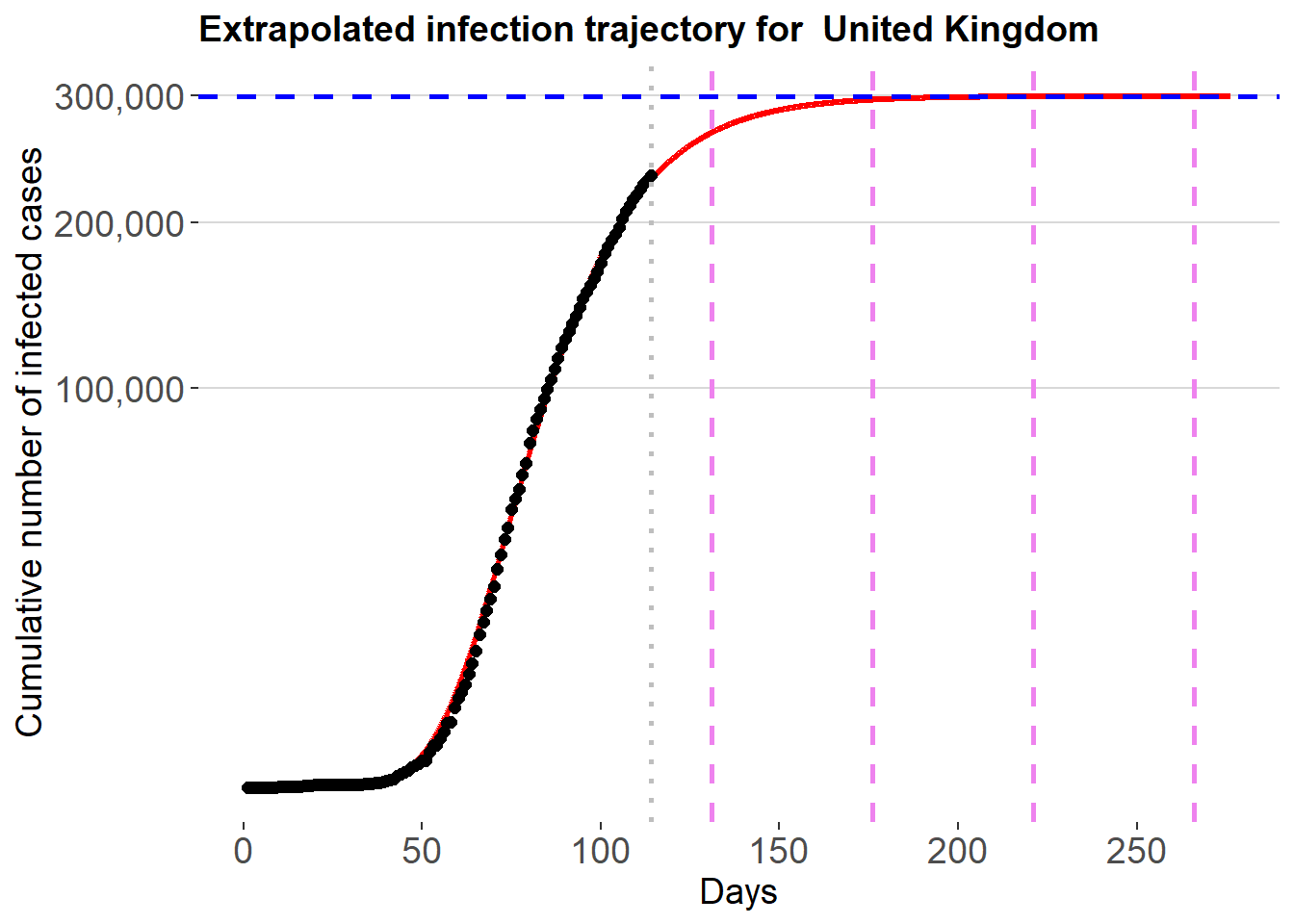

Supplement: S1 File — (ZIP) [file pone.0236860.s005.zip › S1_File/Estimation_of_COVID-19_spread_curves_files/figure-html/unnamed-chunk-10-1.png]

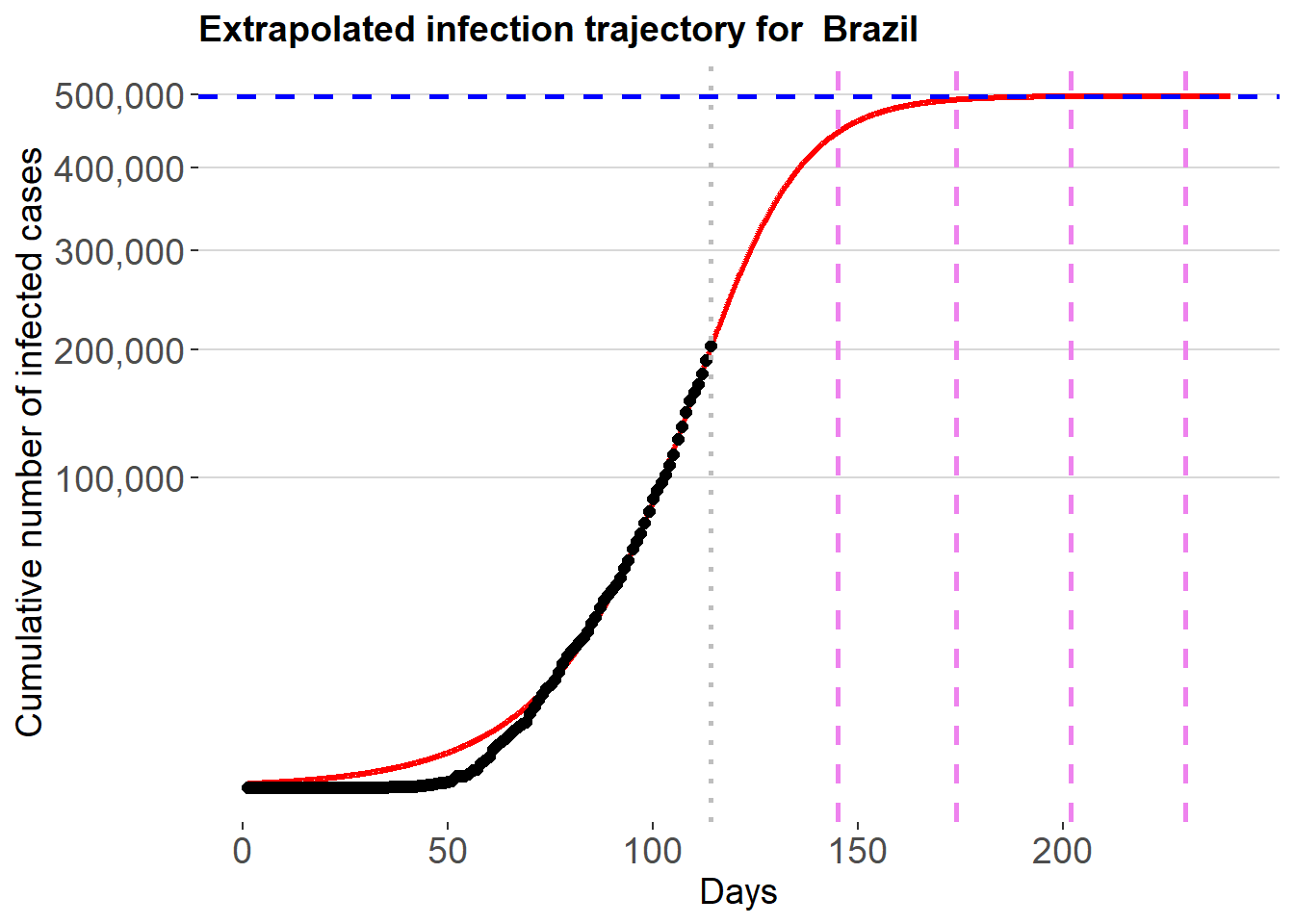

Supplement: S1 File — (ZIP) [file pone.0236860.s005.zip › S1_File/Estimation_of_COVID-19_spread_curves_files/figure-html/unnamed-chunk-13-1.png]

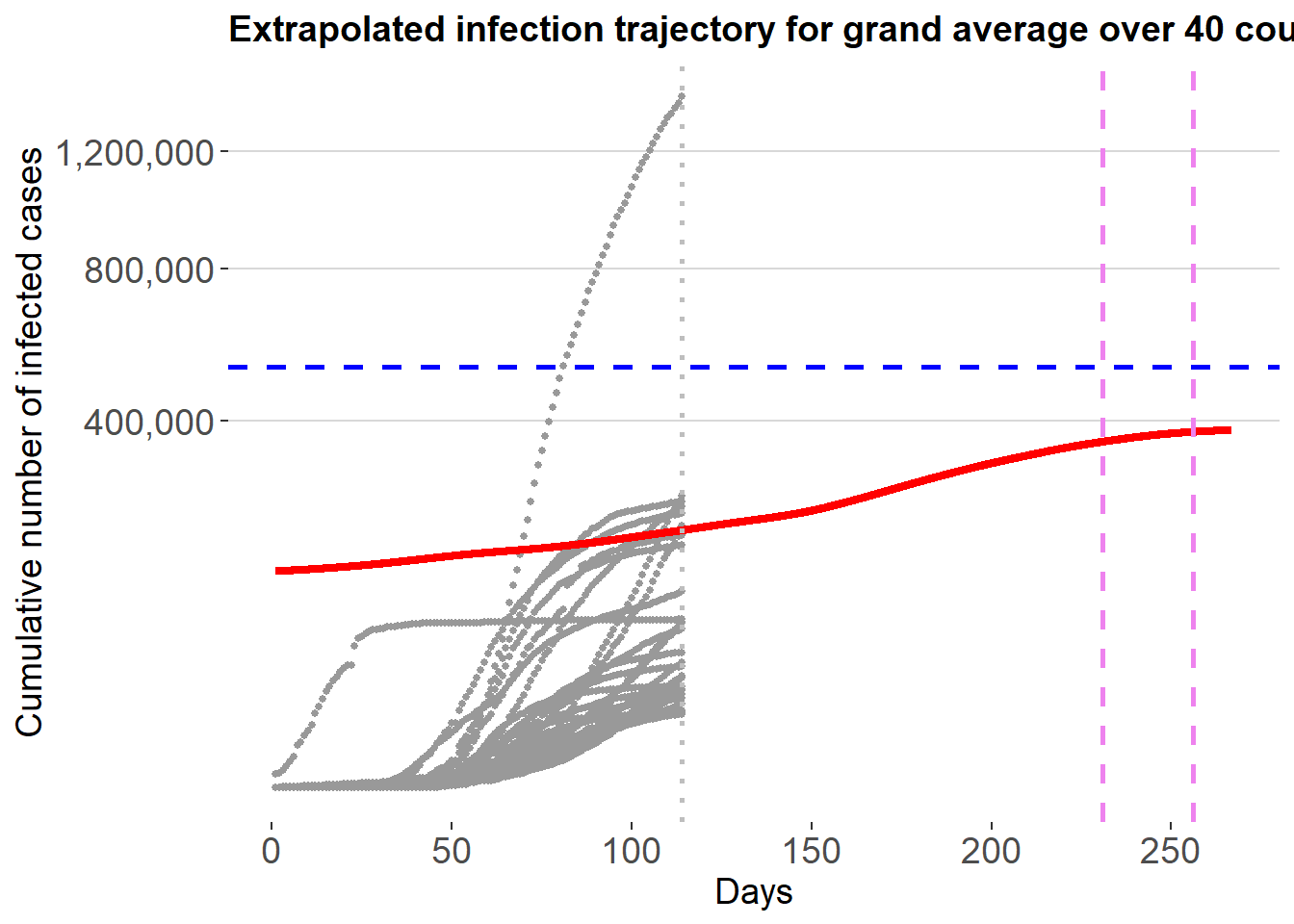

Supplement: S1 File — (ZIP) [file pone.0236860.s005.zip › S1_File/Estimation_of_COVID-19_spread_curves_files/figure-html/unnamed-chunk-16-1.png]

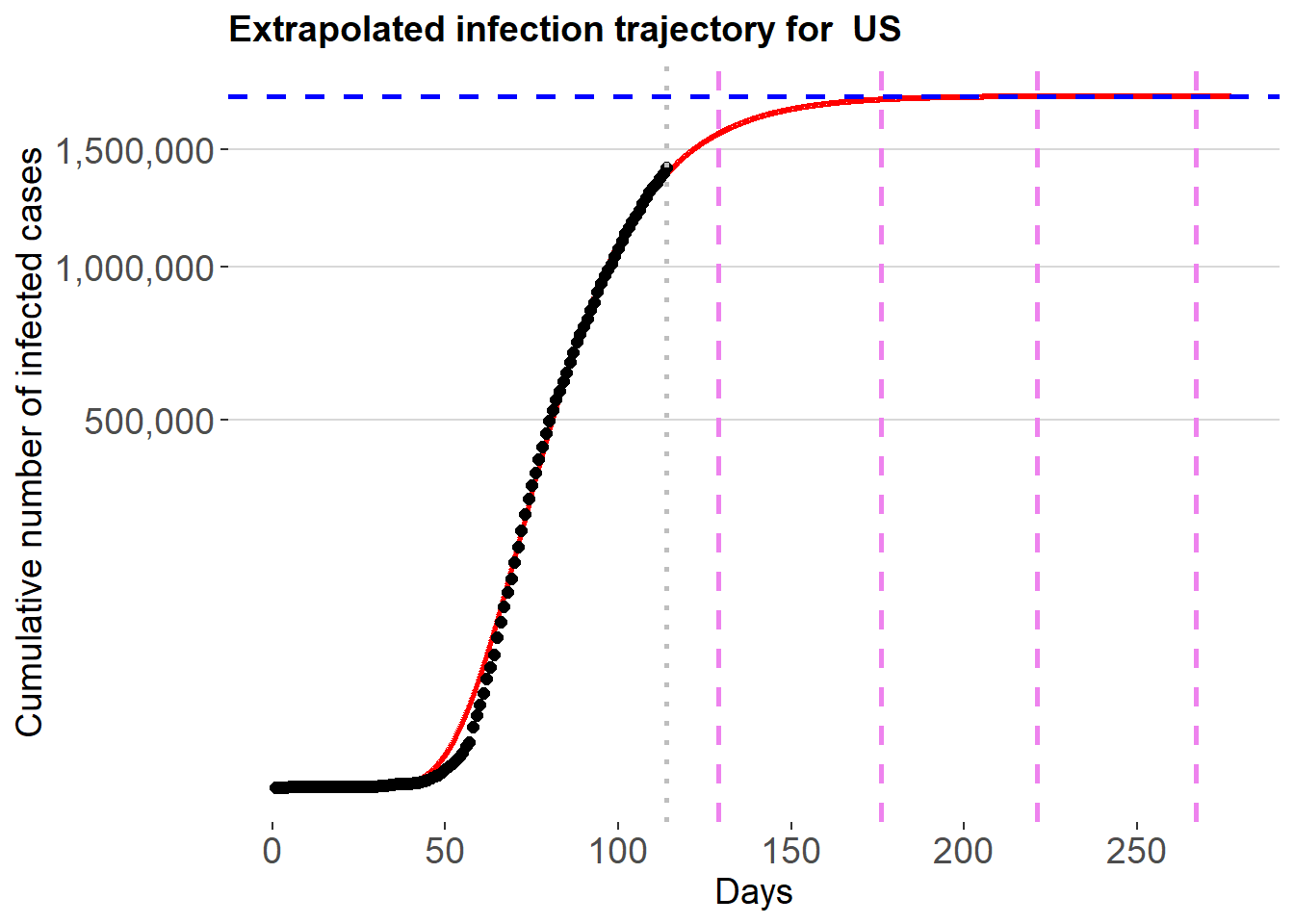

Supplement: S1 File — (ZIP) [file pone.0236860.s005.zip › S1_File/Estimation_of_COVID-19_spread_curves_files/figure-html/unnamed-chunk-4-1.png]

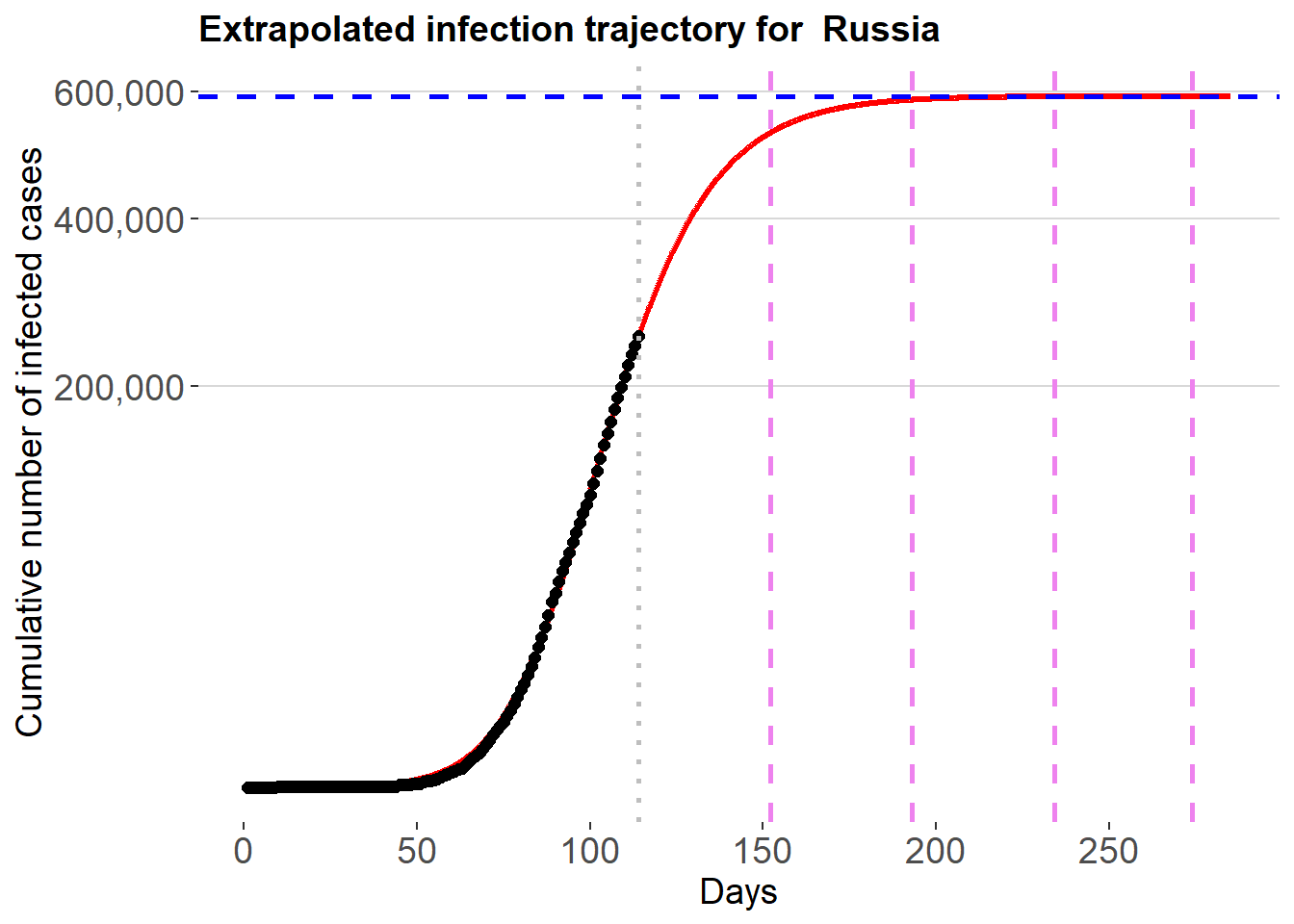

Supplement: S1 File — (ZIP) [file pone.0236860.s005.zip › S1_File/Estimation_of_COVID-19_spread_curves_files/figure-html/unnamed-chunk-7-1.png]
