# Supplementary material for: Estimation of COVID-19 spread curves integrating global data and borrowing information
Source: PLoS One. 2020 Jul 29;15(7):e0236860. doi: 10.1371/journal.pone.0236860 (PMC7390340; doi:10.1371/journal.pone.0236860)
Supplement: Attachment — Submitted filename: Response to Reviewers.docx [file pone.0236860.s006.docx]

Response to reviewers

Dear reviewers,

Thank you so much for your time and comments. The followings are changes we made based on your suggestions.

1. **Manuscript.** We updated the structure of our manuscript to meet PLOS ONE's style requirements.
2. **Manuscript - line 134.** As for your comment on mean squared error (MSE) comparison, we changed the test-days spanning from 2 to 28 days, and also drew a boxplot to compare the MSE in the three models, denoted by $M_{1}$, $M_{2}$ and $M_{3}$.
3. **Manuscript - line 167.** As for your comment on the confidence interval in Fig 5, we described how we obtained the 95% credible intervals for the final epidemic size (that is, parameter $\theta_{1}$).
4. **Manuscript - line 186.** As for your comment on the time to reach the final plateau of the epidemic (which we call flat time point in our manuscript), we changed the definition of flat time point to the time to reach a percentage of the final epidemic size. On the manuscript, we updated results and figures accordingly: Fig 6, Fig 7, Fig 8, and S5 Appendix (Infection trajectories for the top 20 countries).
5. **Manuscript - line 256 to 264.** As for your comment on Table 1 and corresponding interpretation, we added posterior mean of the coefficients in table. Before the posterior inference, we standardized the design matrix by following a standard rule in variable selection. We mention that the estimated values are more likely to be thought as conceptual measures representing a sensitivity of the response with respect to a change of value of a predictor.
6. **Manuscript - line 329.** As for your comment on the discussion of a limitation of the work, we added relevant discussion in the Section Discussion.
7. **S1 Appendix: Research data.** As for your comment on the selection of the 40 countries, we explained why we chose the 40 countries in our research.
8. **S1 Appendix - Table S.2.** As for your comment on the imprecision in part of the data, we added a note about possible bias caused by these data.
9. **S1 File.** As we comply with journal requirement, we share our minimal dataset and codes that can be utilized in a standalone way.
